# Genetic Characterization and Pathogenicity of a Novel Recombined Porcine Reproductive and Respiratory Syndrome Virus 2 among Nadc30-Like, Jxa1-Like, and Mlv-Like Strains

**DOI:** 10.3390/v10100551

**Published:** 2018-10-09

**Authors:** Long Zhou, Runmin Kang, Jifeng Yu, Bo Xie, Changying Chen, Xingyu Li, Jing Xie, Yonggang Ye, Lu Xiao, Jinling Zhang, Xin Yang, Hongning Wang

**Affiliations:** 1 School of Life Science, Sichuan University, Animal Disease Prevention and Food Safety Key Laboratory of Sichuan Province, Key Laboratory of Bio-resources and Eco-environment, Ministry of Education, Chengdu 610064, Sichuan, China; 2014322040041@stu.scu.edu.cn (L.Z.); xinyang@scu.edu.cn (X.Y.); 2 Sichuan Animal Science Academy, Sichuan Provincial Key laboratory of Animal Breeding and Genetics, Chengdu 610066, Sichuan, China; angelina_0708@hotmail.com (R.K.); yujifeng2009@sohu.com (J.Y.); xinyue199@aliyun.com (X.L.); xiemm75@163.com (J.X.); yeyg0202@sina.com (Y.Y.); yaanxiaolu@163.com (L.X.); m13084448812_1@163.com (J.Z.); 3 Chengdu Chia Tai Agro-industry & Food, Animal healthy disease service, Chengdu 610081, Sichuan, China; bobosky123@163.com (B.X.); ccyenanging@163.com (C.C.)

**Keywords:** porcine reproductive and respiratory syndrome virus (PRRSV), modified live virus (MLV), recombination, mutation, pathogenicity

## Abstract

Recombination among porcine reproductive and respiratory syndrome viruses (PRRSVs), coupled with point mutations, insertions, and deletions occurring in the genome, is considered to contribute to the emergence of new variants. Here, we report the complete genome sequences of a PRRSV field strain, designated SCN17, isolated from a RespPRRS MLV-vaccinated piglet in China in 2017. Sequence alignment revealed that SCN17 had discontinuous 131-amino acid (111 + 1 + 19-aa) deletion in the NSP2-coding region identical to that of NADC30 when compared to VR-2332. Notably, the strain, SCN17, contained an additional 1-aa deletion in NSP2, a 1-aa deletion in ORF5, and a unique 3-nt deletion in the 3′-UTR. Phylogenetic analysis showed that SCN17 clustered into NADC30-like lineage based on ORF5 genotyping, whereas it belonged to an inter-lineage between the NADC30-like and VR-2332-like lineages as established based on the full-length genome. Importantly, the SCN17 was identified as a novel virus recombined between a NADC30-like (moderately pathogenic), a JXA1-like (highly pathogenic), and an attenuated vaccine strain, RespPRRS MLV (parental strain VR-2332). Furthermore, we tested its pathogenicity in piglets. SCN17 infection caused a persistent fever, moderate interstitial pneumonia, and increased the viremia and antibody levels in the inoculated piglets. Of note, all SCN17-infected piglets survived throughout the study. The new virus was showed to be a moderately virulent isolate and have lower pathogenicity than HP-PRRSV strain, SCwhn09CD. Our results provide evidence for the continuing evolution of PRRSV field strain by genetic recombination and mutation leading to outbreaks in the vaccinated pig populations in China.

## 1. Introduction

Porcine reproductive and respiratory syndrome (PRRS) is a viral disease that has led to serious economic consequences in the global swine industry since it was first discovered in 1987 in the United States [1]. The etiological agent of this disease, the PRRS virus (PRRSV), is an enveloped virus that contains a single-stranded, positive-sense RNA genome of 15.1–15.5 kilobases (kb) [2]. In the latest taxonomy of viruses, PRRSV has been classified in the genus, *Porartevirus*, of the family, *Arteriviridae*, order, *Nidovirales* [2,3]. The 5′ three-quarters (ORF1a and ORF1b) of the PRRSV genome encode two replicase polyproteins (pp1a and pp1b) that contain proteins necessary for RNA replication. In contrast, the 3′-end of the remaining encoding genome (ORFs 2–7) encodes eight viral structural proteins, including the minor envelope proteins (GP2a, E, GP3, GP4, and GP5a), the major envelope proteins (GP5 and M), and the nucleocapsid protein (N) [4,5,6,7]. To date, two distinct types of PRRSV have been identified worldwide, namely PRRSV-1 (European origin, prototype strain Lelystad virus, LV) and PRRSV-2 (North American origin, prototype strain, VR-2332). Although the two types of PRRSV produce similar clinical symptoms and pathological changes in the infected pigs, they differ significantly in their antigenic properties and genetic content [6,8,9].

In China, PRRSV-2 is highly predominant in the field and has been a major disease problem for the swine industry over the past 20 years. Based on the global PRRSV classification system, the abundance of PRRSV-2 strains in China can be divided into four lineages: JXA1-like/CH-1a-like (lineage 8), VR-2332-like (lineage 5), QYYZ-like (lineage 3), and NADC30-like (lineage 1) [10,11]. The recombination among PRRSVs is an attribute of the virus family that is considered to contribute to the emergence of new PRRSV variants [12]. Since the QYYZ-like strains emerged in China in 2010 [13] and the NADC30-like strains in 2013 [14], extensive genetic recombination among members of different lineages has been frequently reported, and the recombinants are becoming more common in the field. Recently, QYYZ-like PRRSV strains were discovered to recombine with JXA1-like strains (GDsg and GD1404) [15,16]. Additionally, NADC30-like strains were found to recombine with JXA1-like strains (HENAN-HEB, JL580, FJ1402, TJnh1501, 15HEN1, HNhx, and SCnj16) [17,18,19,20,21,22,23] or VR-2332-like strains (Chsx1401, HENAN-XINX, and HNyc15) [14,20,24]. Moreover, several complex recombinants among the three lineages of PRRSV-2 strains (SDhz1512, SD17-38, SCcd16, SCcd17, and SCya17) have also been observed in China [25,26,27,28].

Previous studies have revealed that natural recombination can occur between PRRSV field strains as well as between field and modified live virus (MLV) vaccine strains [22,29,30]. Since the highly pathogenic PRRS (HP-PRRS) outbreaks in China in 2006 [31], imported and domestic commercial MLV vaccines, such as CH-1R (parental strain CH-1a), Ingelvac PRRS MLV/RespPRRS MLV (VR-2332), JXA1-P80 (JXA1), TJM-F92 (TJ), HuN4-F112 (HuN4), R98 (R98), and GDr180 (GD), have been used to safeguard against PRRSV-2 throughout the country. Despite the intensive vaccination, the control of the disease remains difficult. PRRSV-2 variants and vaccine-like viruses have been frequently identified under field conditions [13,19,22,29]. Recently, NADC30-/QYYZ-like strains circulating in the field were reported to recombine with Chinese HP-PRRSV-derived MLV vaccines (JXA1-P80, TJM-F92-like) [16,19], leading to outbreaks of clinical disease on pig farms. In the present study, a novel recombinant PRRSV strain (SCN17) was isolated from a pig farm practicing MLV vaccination in the Sichuan Province, China, in 2017. The newly emerged PRRSV isolate is likely a product of multiple recombination events among three different PRRSV strains, namely, the NADC30-like, JXA1-like, and RespPRRS MLV-like vaccine (an attenuated vaccine developed from the North American strain, VR-2332), which has never been described. Meanwhile, multiple unique point mutations and deletions occurred in its genome. Furthermore, in an animal challenge study, we discovered that it was moderately pathogenic to piglets. Our results indicate that both recombination and mutations are involved in the evolution of PRRSV in the field.

## 2. Materials and Methods

### 2.1. Sample Collection and Virus Isolation

The PRRSV strain, designated SCN17, was propagated in primary porcine alveolar macrophage cells (PAMs) inoculated with suspensions of lung tissue homogenates from diseased piglets that were collected at a pig farm in the Sichuan area, China, in 2017. The affected pregnant sows experienced abortions and stillbirths. The affected piglets had high fevers (>40 °C) and exhibited overt signs of respiratory illness, with approximately 3.7% mortality. RT-PCR was used to confirm PRRSV positivity in suspensions of lung tissue homogenates, which were then inoculated into PAMs. The PAMs were maintained in RPMI-1640 medium (Transgene, Beijing, China) supplemented with 10% fetal bovine serum (HyClone, Logan, UT, USA) at 37 °C in a humidified 5% CO_2_ atmosphere as described previously [23]. Indirect immunofluorescence assay (IFA) was performed to detect viral antigens in PAMs using GTX129270 (a monoclonal antibody against PRRSV N protein; GeneTex Inc., Irvine, CA, USA). It is noteworthy that SCN17 was isolated from a 6-week-old piglet that had been vaccinated with a commercially available, live attenuated, RespPRRS MLV vaccine (Boehringer Ingelheim, Germany). The pig was initially vaccinated seven days after birth and received a booster vaccination at 21 days. The SCN17 strain was passaged twice in PAMs and purified by plaque assay. Then, the purified viruses were further used for full-genome sequencing.

### 2.2. RNA Extraction and Full-Genome Sequencing

The viral genomic RNA was extracted from PRRSV SCN17-infected PAMs using the TRIzol reagent (Invitrogen, Carlsbad, CA, USA) according to the manufacturer’s instructions. cDNA was generated using a PrimeScript^TM^ RT reagent Kit (TaKaRa, Dalian, China) following the supplier’s guidelines. Twelve pairs of overlapped primers specific for the PRRSV-2 genome were utilized for amplifying the complete genome of SCN17, as described previously [23]. Further, the purified RT-PCR products were ligated to a pMD19-T cloning vector (TaKaRa, Dalian, China), and at least three independent recombinant clones were sequenced by Sangon Biological Technology (Beijing, China). The 5′- and 3′-terminal sequences of the viral genome were determined using a SMARTer RACE 5′/3′ kit (TaKaRa, Dalian, China) following the manufacturer’s protocol. Finally, the sequences of fourteen overlapping fragments from SCN17 were assembled into full-length consecutive sequences.

### 2.3. Genome Alignment and Phylogenetic Analysis

The obtained full-length genomic sequences of SCN17 were assembled using the SeqMan program of DNAstar software, version 7.0 (DNASTAR Inc., Madison, WI, USA). Further, the sequences of PRRSV genomes, ORFs, and deduced proteins were analyzed by the EditSeq and MegAlign programs of DNAstar (DNASTAR Inc., Madison, WI, USA). Next, phylogenetic analysis was performed based on the nucleotide sequences, generated by the distance-based neighbor-joining method using the Molecular Evolutionary Genetics Analysis 6 (MEGA 6) software (www. megasoftware.net). Finally, the bootstrap values of the phylogenetic tree were evaluated with 1000 replicates.

### 2.4. Recombinant Analysis of Strain SCN17

To detect potential recombination events in the genome of the newly emerged PRRSV field strain, SCN17, its complete genome sequences as well as those of other representative strains were subjected to recombination screening using the Recombination Detection Program 4 (RDP4, v4.24) software implementing seven methods (RDP, Bootscan, GENECONV, MaxChi, Chimaera, SiScan, and 3Seq) [32]. Default parameters were utilized and the recombination events were considered significant when confirmed by at least five of the aforementioned methods. To visualize the recombination events, a similarity analysis was implemented in the SimPlot software (v3.5.1, Baltimore, MD, USA) with a 200-bp window and a 20-bp step. Furthermore, a series of phylogenetic trees based on each of the recombinant fragments were constructed to confirm these putative recombination events.

### 2.5. Pathogenicity Analysis of Strain SCN17

Fourteen 4-week-old healthy Large White piglets were obtained from the conservation farm of Tibetan pigs, Sichuan Animal Science Academy (Chengdu, Sichuan, China). The piglets were confirmed to be not infected with PRRSV, classical swine fever virus (CSFV), pseudorabies virus (PRV), and porcine circovirus type 2 (PCV2) by PCR or RT-PCR. All animals were randomly divided into three groups: SCwhn09CD-inoculated group (*n* = 5), SCN17-inoculated group (*n* = 5), and uninfected RPMI-1640 medium-inoculated control group (*n* = 4), and maintained in individual rooms. SCwhn09CD (GenBank no. JN836553) is a HP-PRRSV strain isolated in the Sichuan area in 2009 [33]. Each piglet in each of the groups was intranasally administered with 2 mL (2 × 10^5^ TCID_50_/mL) of virus SCN17, SCwhn09CD, and RPMI-1640 medium. The health conditions of the pigs were carefully monitored throughout the experimental period. The clinical presentations, pig death, and rectal temperatures of the inoculated piglets were recorded daily. For detection of PRRSV-specific antibodies, serum samples were collected on 0, 3, 5, 7, 10, and 14 days post-inoculation (dpi) and subjected to tests using a commercial enzyme-linked immunosorbent assay (ELISA) kit, IDEXX HerdChek ELISA (Westbrook, ME, USA). PRRSV viral RNA in the serum of the inoculated pigs was determined by one-step Taq-Man RT-qPCR. All pigs were humanely euthanized at 14 dpi. At necropsy, lung samples were collected and immediately fixed in 10% neutral buffered formalin for histopathology analysis.

The virus challenge experiment was approved by the Animal Ethics Committee (AEC) of the College of Life Sciences, Sichuan University (12 May 2018, SYXK-Chuan-2013-185). All experimental procedures and animal care strictly followed the guidelines of Animal Management of Sichuan University.

### 2.6. Statistical Analysis

Statistical analysis of the differences in the body temperature, weight gain, virus copies in the serum samples, and ELISA antibody levels was performed using one-way ANOVA with a Tukey’s *t*-test in GraphPad Prism software 5.0 (GraphPad Software Inc., San Diego, CA, USA). Differences were considered statistically significant at a *p*-value of <0.05. All data were expressed as mean ± standard deviations (SD).

## 3. Results

### 3.1. Full-Genome Characterization and Homology Analysis

The complete genome sequence of the strain, SCN17, was assigned into the GenBank sequence database under the accession number, MH078490. The complete genomic sequence of SCN17 is 15,010 nucleotides (nt) in length, with a 189-nt 5′-UTR and a 147-nt 3′-UTR, excluding the poly (A) tail at the 3′ end. Homology analyses revealed that the strain, SCN17, shares the highest identity with the U.S. strain, NADC30 (92.3%, lineage 1), and have 86.7%, 83.4%, 82.9%, and 80.3% identity with the classical North American strain, VR-2332 (lineage 5), Chinese classical strain, CH-1a (lineage 8), HP-PRRSV strain, JXA1 (lineage 8), and QYYZ (lineage 3), respectively. Additionally, SCN17 had slightly higher homology to the MLV vaccine strain, RespPRRS MLV (86.8%), than to its parental strain, VR-2332 (86.7%) (Table 1).

Each region of the SCN17 genome was further compared with those of NADC30, VR-2332, JXA1, CH-1a, QYYZ, and the MLV vaccine strain (RespPRRS MLV). As can be seen in Table 1, thirteen regions of SCN17 shared higher homology at the nucleotide level (91.6%–96.5%) and identity at the amino-acid level (91.5%–100%) with NADC30, including nsp1β, 2, 7a, 7b, 8, 11–12, ORF2a, 2b, 3–4, and 6–7. Notably, six regions of SCN17 exhibited higher nucleotide homology (96.4%–100%) and amino-acid identity (95.7%–100%) with the MLV vaccine strain, RespPRRS MLV, including nsp1α, 4–6, and 9–10, whereas four regions exhibited relatively higher nucleotide homology (96.4%–100%) and amino-acid identity (98.3%–100%) with VR-2332, including nsp1α, 4, 6, and 9. In addition, the nsp3 region of SCN17 shared higher nucleotide homology (92.0%) with VR-2332 and RespPRRS MLV, whereas it exhibited higher amino-acid identity (96.5%) with NADC30. The ORF5 region displayed higher nucleotide homology (90.4%) with JXA1, whereas it had exhibited higher amino-acid identity (89.6%) with NADC30. No fragment of SCN17 had higher nucleotide homology or amino-acid identity with QYYZ (Table 1). These findings indicated that the genome of SCN17 is closely related to that of the MLV vaccine strain, RsepPRRS MLV, and recombination might occur in its genome.

### 3.2. Sequence Analysis of Nsp2, GP5, and 3′-UTR

**Figure 1:**
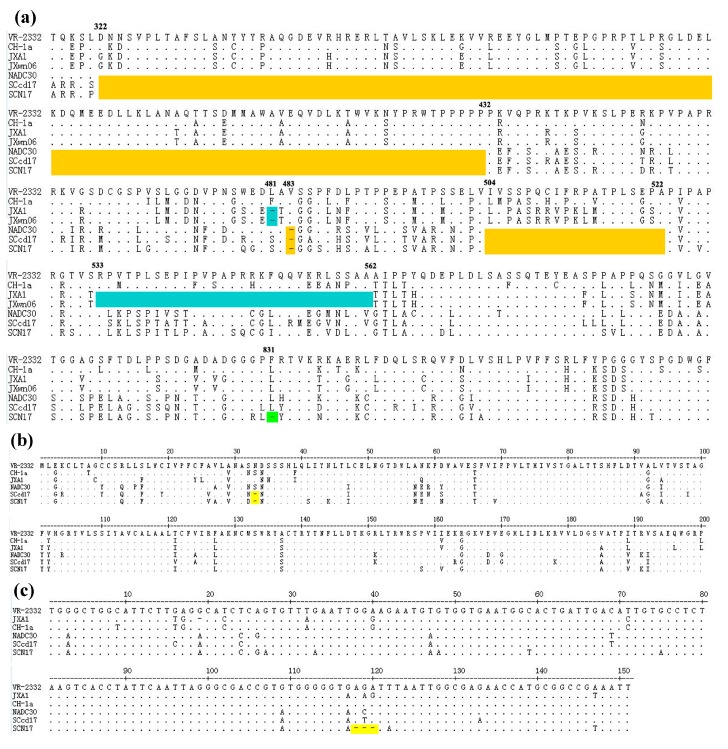
Multiple sequences alignment of NSP2, GP5, and 3′-UTR from representative PRRSV strains and SCN17 isolate. (**a**) Three discontinuous amino acid deletions at positions 322–432, 483, and 504–522 (yellow regions), and an additional 1-aa deletion at position 831 (green region) in NSP2-cording region are observed in SCN17. The blue regions (positions 481, 553–562) indicate the classical HP-PRRSV deletion in NSP2-cording region; (**b**) one amino acid deletion in GP5 at position 33 of SCN17 and SCcd17 (yellow region); (**c**) three continuous nucleotides deletion in 3′-UTR at positions 118 to 120 of SCN17 (yellow region). Five representative PRRSVs, including NADC30 (JN654459), JXA1 (EF112445), JXwn06 (EF641008), VR-2332 (AY150564), and CH-1a (AY032626), are included in the analysis.

The amino-acid alignment of the NSP2 of SCN17 with the other strains revealed that the new PRRSV isolate had the same amino-acid (aa) deletions as NADC30 (first identified in the USA in 2008) [34]. These deletions were identified as a discontinuous 131-aa deletion (111-aa from positions 323 to 433, 1-aa at position 481, and 19-aa at positions from 533 to 551) when compared with the sequence of VR-2332 (Figure 1a). Moreover, the amino-acid alignment of SCN17 GP5 with that of the other strains showed that SCN17 had a 1-aa deletion at position 33 (Figure 1b), which we had previously reported for the genome of SCcd17 (MG914067) [27]. Meanwhile, SCN17 had an additional 1-aa deletion at position 831 in the NSP2-cording region and a novel continuous 3-nt deletion in its 3′-UTR at positions from 118 to 120 (Figure 1c), which had not been previously reported. These data suggested that multiple point deletions occurred in the genome of SCN17.

### 3.3. Phylogenetic Analysis

**Figure 2:**
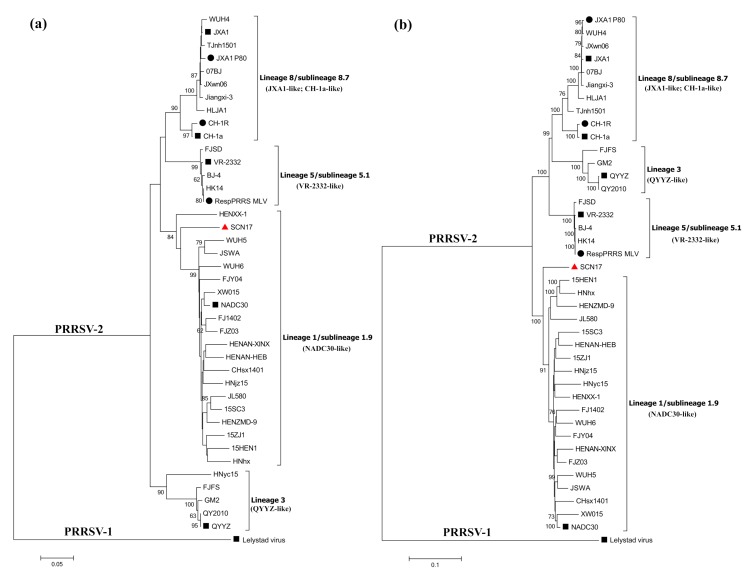
Phylogenetic trees based on ORF5 (**a**) and complete genomic sequences (**b**) of strain SCN17 and 40 reference PRRSV strains. The new recombinant virus, SCN17, is marked with a “red triangle”. PRRSV-2 strains in China are clustered into four lineages (lineages 1, 3, 5, and 8), and the representative strains of each lineage are marked with “black squares”, while the MLV vaccines were labeled with “black circles”. The phylogenetic trees are computed using the neighbor-joining method with 1000 replicates using the MEGA6 program. Scale bar indicates nucleotide substitute per site.

Phylogenetic trees were generated based on the ORF5 and full complete nucleotide sequences of SCN17, along with another 40 representative PRRSV strains available in GenBank, including PRRSV-2 isolates obtained during 1996–2016 from China (*n* = 36), North America (*n* = 3), and PRRSV-1 from Europe (*n* = 1) (Supplementary Table S1). As can be seen in Figure 2a, the prevalent PRRSV-2 strains in China could be divided into four lineages based on the ORF5 sequences: NADC30-like (lineage 1/1.9), QYYZ-like (lineage 3), VR-2332-like (lineage 5/5.1), and JXA1-like/CH-1a-like (lineage 8/8.7). Notably, strain SCN17 was classified in the NADC30-like lineage based on the ORF5 genotyping, whereas it was clustered into a separate branch of the phylogenetic tree according to its full-length genome, which was located between lineage 1, represented by NADC30, and lineage 5, represented by VR-2332 (Figure 2b). These findings suggested that a possible recombination had occurred between different PRRSV lineages shaping the genome of SCN17.

### 3.4. Recombination Analysis of SCN17

**Figure 3:**
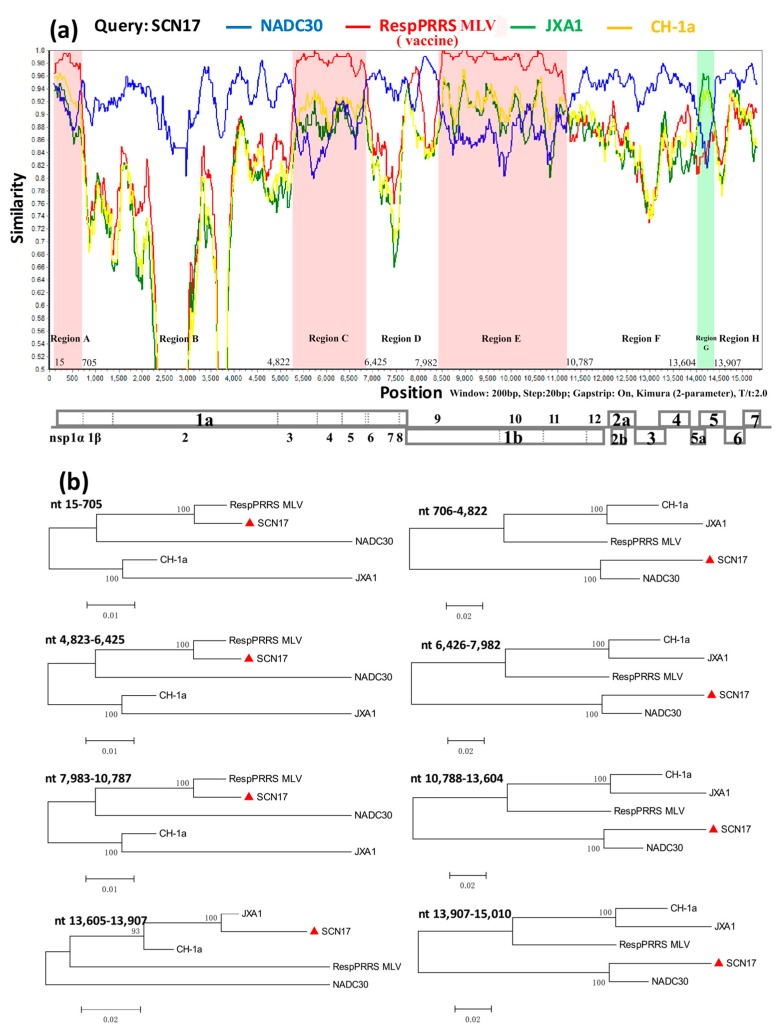
Genome recombination analyses of the PRRSV isolate, SCN17. The *y*-axis indicates the percentage similarity between the query sequence (SCN17) and four representative sequences. (**a**) Genome scale similarity comparisons of SCN17 with NADC30 (blue), RespPRRS MLV (red), JXA1 (green), and CH-1a (yellow). The eight supposed recombination regions (regions A-H) and the recombination breakpoints are shown at the bottom with nucleotide sites. A complete viral genome structure is shown under the similarity plot with reference to VR-2332; (**b**) phylogenetic trees based on each recombinant region (region A-H) of SCN17.

To identify the possible recombination events, we detected the recombination using RDP4 and SimPlot software. Considering that the PRRSV strain, SCN17, was isolated from a RespPRRS MLV-vaccinated piglet and was closely related to the vaccine strain, RespPRRS MLV, as established by genome homology analysis (Table 1), we set SCN17 as the query sequence, and NADC30, RespPRRS MLV, JXA1, and CH-1a as the reference sequences. The analysis results revealed that SCN17 had remarkably high degrees of certainty (*p*-values ≤ 1 × 10^−6^) in the results of at least five detection methods (Table 2). Strikingly, eight potential recombination breakpoints were identified from the whole genomic sequences alignment, which were located in 5′-UTR (nt 15), nsp1β (nt 705), nsp3 (nt 4822), nsp7 (nt 6425), nsp9 (nt 7982), nsp11 (nt 10,787), ORF5 (nt 13,604), and ORF5 (nt 13,907) (Figure 3a). These breakpoints in SCN17 separated its genome into eight regions, where regions A, C, and E (nt 15–705, 4822–6425, and 7983–10,787, respectively) were closely related to the RespPRRS MLV-like strain, whereas regions B, D, F, and H (nt 706–4822, 6426–7982, 10,788–13,604, and 13,907–15,010, respectively) were closely associated with the NADC30-like strain, and region G (nt 13,605–13,907) was tightly linked to the JXA1-like strain (Figure 3b). The analysis revealed that SCN17 had likely originated from multiple recombination events among NADC30-like (lineage 1), JXA1-like (lineage 8), and the vaccine strain, RespPRRS MLV (lineage 5).

### 3.5. Pathogenicity Analysis of SCN17

#### 3.5.1. Clinical Symptoms

**Figure 4:**
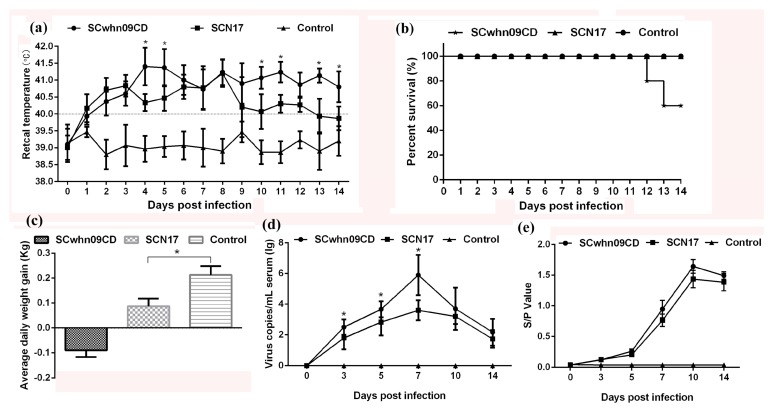
The rectal temperature, survival rate, weight gain, viremia, and antibody level of piglets during the challenge study. (**a**) Rectal temperatures of piglets inoculated with SCwhn09CD, SCN17, and RPMI-1640 medium. Mean ± SD (error bars) temperatures (°C) are shown. The fever cut off value was set at 40.0 °C; (**b**) the survival and mortality curves of the inoculated piglets; (**c**) average daily weight gain of the inoculated piglets. Mean ± SD (error bars) weight gain (kg) for the survival pigs of each group is shown; (**d**) the PRRSV RNA copies in sera of pigs at different days post challenge were detected by one-step Taq-Man RT-qPCR. Mean ± SD (error bars) viral RNA copies (lg copies/mL) are shown; (**e**) PRRSV-specific antibodies in sera of pigs at different days post challenge. Pig serum was assayed for PRRSV-specific antibodies using ELISA kit, IDEXX HerdCheck ELISA. ORF5 gene was amplified with specific primers, the sense primer was 5’-AGCCTGTCTTTTTGCCATTCT-3’, and the reverse primer was 5’-CTTTTGTGGAGCCGTGCTATC-3’. Mean ± SD (error bars) S/P values (lg copies/mL) are shown. The significant difference is marked with the asterisk (*).

After challenge, SCwhn09CD-infected pigs exhibited slight clinical signs within 2–3 dpi, such as febrility, cough, sneezing, and anorexia. More severe clinical symptoms, including high fever, shivering, hyperspasmia, and respiratory distress, were manifested within 4–14 dpi. By contrast, the clinical manifestations were milder in SCN17-infected pigs: Persistent fever within 1–12 dpi, anorexia, dyspnea, and cough. Two out of five SCwhn09CD-challenged piglets died on 12 and 13 dpi, respectively, whereas all SCN17-infected and RPMI-1640-inoculated pigs survived until the end of study (Figure 4b). Pigs infected with the HP-PRRSV strain, SCwhn09CD, showed a febrile response (39.9 °C) starting from 1 dpi, which lasted for 13 consecutive days (40.3–41.4 °C). The pigs infected with SCN17 had an early febrile peak at 3 dpi and a second peak at day 8 post-infection; they had average rectal temperatures that rose above 40 °C within 2–12 dpi. The average rectal temperature of the SCN17-infected pigs was significantly lower than that of the pigs infected with SCwhn09CD at 4, 5, 10, 11, 13, and 14 dpi (*p* < 0.05). The average rectal temperature of the RPMI-1640-inoculated pigs remained below 40 °C throughout the experiment (Figure 4a). The SCwhn09CD-infected pigs lost body weight (−0.09 kg) more rapidly than the SCN17-infected and RPMI-1640-inoculated pigs. In contrast, the SCN17-infected pigs and RPMI-1640-inoculated pigs average daily gains of 0.087 kg and 0.213 kg, respectively, and there was a significant difference between the two groups (*p* < 0.05) (Figure 4c). These results indicated that SCN17 is less pathogenic than the HP-PRRSV strain, SCwhn09CD.

#### 3.5.2. Viremia and PRRSV-Specific Antibodies

Viremia of piglets in the two PRRSV-infected groups was detected within 3–14 dpi. As illustrated in Figure 4d, the serum virus RNA copy numbers in the SCN17-infected pigs were significantly lower than those in the SCwhn09CD-infected pigs during 3–7 dpi (*p* < 0.05). The viral titers of SCwhn09CD- and SCN17-infected groups both reached peak levels at 7 dpi. No viremia was detected in the serum samples from the RPMI-1640-inoculated pigs throughout the entire experimental period. Meanwhile, the serum levels of PRRSV-specific antibodies were evaluated by ELISA. Lower levels of PRRSV-specific antibodies were found in SCN17-infected pigs than in SCwhn09CD-infected pigs during 5–14 dpi; however, no statistically significant differences were noted. The antibodies in the control group remained negative throughout the experiment (Figure 4e). Additionally, the viruses were recovered from the sera of pigs in the SCwhn09CD- and SCN17-challenge groups, and the ORF5 gene was sequenced to confirm they were the original viruses.

#### 3.5.3. Macroscopic and Microscopic Lesions in the Lungs

**Figure 5:**
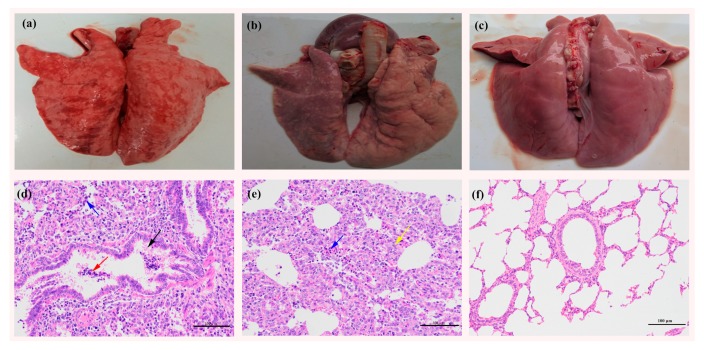
Gross and microscopic lung lesions observation of the inoculated piglets. (**a**) Severe interstitial pneumonia with hemorrhage, consolidation, and pulmonary edema were observed in SCwhn09CD-inoculated pigs; (**b**) moderate interstitial pneumonia with consolidation was observed in SCN17-inoculated pigs; (**c**) no gross lung changes were observed in the RPMI-1640-inoculated pigs; (**d**) interstitial pneumonia with infiltration of inflammatory cells, foci of necrosis (blue arrow), necrotic cell mass (red arrow), and exfoliated epithelial cells (black arrow) in the bronchiole could be observed in SCwhn09CD-inoculated pigs; (**e**) interstitial pneumonia with infiltration of inflammatory cells, foci of necrosis (blue arrow), and alveolar septal thickening (yellow arrow) could be observed in SCN17-inoculated pigs; (**f**) no pathological lesions were identified in the RPMI-1640-inoculated pigs. Original magnification, 200×.

The major gross pathological lesions in the lungs of PRRSV-challenged pigs were characterized by interstitial pneumonia. Severe interstitial pneumonia with hemorrhage, consolidation, and pulmonary edema was found in the SCwhn09CD-challenged pigs (Figure 5a), whereas moderate interstitial pneumonia with consolidation and pulmonary edema was observed in the SCN17-challenged pigs (Figure 5b). Microscopic examination revealed that SCwhn09CD-challenged pigs displayed interstitial pneumonia with infiltration of inflammatory cells, foci of necrosis (blue arrow), necrotic cell mass (red arrow), and exfoliated epithelial cells (black arrow) in the bronchiole (Figure 5d). The lung sections of SCN17-challenged pigs developed interstitial pneumonia with infiltration of inflammatory cells, foci of necrosis (blue arrow), and alveolar septal thickening (yellow arrow) (Figure 5e). No macroscopic and microscopic pathological lesions were detected in the lungs of RPMI-1640-inoculated pigs (Figure 5c,f).

## 4. Discussion

PRRSV is highly prevalent in pig populations and is responsible for severe economic losses to the swine industry worldwide. Similarly, to other RNA viruses, PRRSV has the ability to continuously undergo genetic/antigenic changes [35]. The poor replication fidelity of viral polymerase, point mutation, recombination, and immune pressure selection are considered the underlying molecular mechanisms for generating viral variability and diversity, which promote the evolution of PRRSV [10,36]. Since the HP-PRRSV strain (represented by JXA1) was first reported in China in 2006 [31], the HP-PRRSV-like viruses have always been accompanying the Chinese swine industry. Within the succeeding years, the HP-PRRSVs with a 30-aa deletion in NSP2 became the dominant strains circulating in China [37,38,39,40]. However, with the emergence of the QYYZ-like and NADC30-like strains in mainland China in 2010 [13] and 2013 [14], respectively, the diversity of RPRSV remarkably expanded, which further complicated the epidemic situation in the field. Meanwhile, varieties of MLV vaccines were widely used throughout the country, which has undoubtedly increased the immune selective pressure in pig herds that has accelerated the evolution of PRRSV in the field [12,39,40].

In this study, a new PRRSV strain, SCN17, was isolated from a commercial pig farm practicing MLV vaccination. The pigs on the farm suffered from clinical symptoms of high fever, dyspnea, and respiratory distress. Sequence alignment and recombination analysis based on the whole genomic sequences revealed that this new isolate is a recombinant virus obtained by multiple recombination events among the strains, NADC30-like (lineage 1), JXA1-like (lineage 8), and the vaccine strain, RespPRRS MLV (lineage 5). Recent evidence has indicated that recombination events occur between wild viruses and highly pathogenic PRRSV-derived MLV vaccine strains [16,19,22]. However, the complex recombination pattern among a NADC30-like, a JXA1-like, and the classical PRRSV-2-derived MLV vaccine strain (RespPRRS MLV) has never been described. In our previous investigation, we found that JXA1-like, NADC30-like, VR-2332-like, and QYYZ-like strains had coexisted in some pig herds in the Sichuan Province since 2016, and the following different recombination patterns were observed in this region: (i) NADC30-like + JXA1-like [23]; (ii)NADC30-like + JXA1-like + VR-2332-like [27]; and (iii) NADC30-like + JXA1-like + QYYZ-like [28]. This evidence thus further supported the possible recombination among the PRRSV strains, NADC30-like and JXA1-like, and the vaccine strain, RespPRRS MLV (VR-2332-like).

The incidence of recombination events between field and MLV vaccine strains warrants attention because the new recombinant virus may lead to changes in PRRSV virulence in pigs. For example, the strain, TJnh1501 (KX510269), was a recombinant virus between the NADC30-like virus and MLV vaccine (TJMF92-like) that was revealed to be an intermediate virulent strain to piglets between the HP-PRRSV strain, JXwn06, and NADC30-like strain, CHsx1401 [19]. The strain, GDsg (KX621003), was obtained as a recombinant virus between the low pathogenic field strain, QYYZ-like virus, and MLV vaccine (JXA1-P80). Challenge experiments with piglets showed that GDsg had higher virulence than its parental strains, and, particularly, the new virus caused serious hemorrhage and microscopic lesions in the brain [41]. In the present study, SCN17 infection caused persistent high fever, moderate interstitial pneumonia, and high viremia and antibody levels in the inoculated piglets. These results indicated that SCN17 is a moderately virulent PRRSV strain with pathogenicity lower than that of the HP-PRRSV strain, SCwhn09CD. Although all SCN17-infected piglets used in our study survived, with a discrepancy with the field result of 3.7% mortality might exist, pigs infected with the PRRSV strain, SCN17, were considered in our study. On the other hand, other pathogens, such as those causing secondary bacterial infections (*Mycoplasma hyorhinis, Haemophilus parasuis, and Escherichia coli.*) or co-infection with immunosuppressing viruses (PCV2) [42], always accompany PRRSV infections, leading to increased mortality under field conditions.

In recent years, NADC30-like PRRSV variants have been prevailing in large areas of China, increasing the number of outbreaks of clinical disease on pig farms vaccinated with MLV vaccines [13,16,19,22]. Moreover, varieties of commercial MLV vaccines, including Ingelvac PRRS MLV, JXA1-R, TJM-F92, and R98, have been used to evaluate the efficacy against the newly emerged NADC30-like recombinant strains (CHsx1401, TJnh1501, and FJ1402) in China. The results demonstrated that these MLV vaccines provide extremely limited cross-protection efficacy against recombinant strain infections [43,44]. Therefore, novel strategies should be considered to develop effective vaccines against PRRSV in the future.

## 5. Conclusions

In summary, a new PRRSV variant, SCN17, was isolated and its whole genome was sequenced and characterized. The results indicate that SCN17 is a natural recombinant strain between NADC30-like (lineage 1), JXA1-like (lineage 8), and the vaccine strain, RespPRRS MLV (lineage 5) that contains multiple novel aa/nt deletions within its genome, which has never been described in the literature so far. Our animal challenge experiments with pigs showed that SCN17 is a moderately virulent strain with lower pathogenicity than that of the HP-PRRSV strain, SCwhn09CD. This study highlights the importance of the continuous surveillance of PRRSV in domestic pigs and the necessity to update the vaccine strategies against newly emerging PRRSV strains in China.

## Figures and Tables

**Table 1:** Nucleotide and amino acid sequence identity (%) of different regions of the strain, SCN17, compared to five representative PRRSV strains (NADC30, VR-2332, JXA1, CH-1a, and QYYZ) and a vaccine strain (RespPRRS MLV).

Genomic Region	NADC30	VR-2332	JXA1	CH-1a	QYYZ	RespPRRS MLV (Vaccine Strain)
	Pairwise % identity (nt/aa)					
Complete genome	**92.3**	86.7	82.9	83.4	80.3	86.8
5′UTR	93.2	**96.9**	91.3	94.4	90.8	**96.9**
nsp1α	91.9/96.1	**98.7/98.3**	91.1/96.1	93.9/**98.3**	89.1/95.4	**98.7/98.3**
nsp1β	**91.6/91.6**	79.5/77.3	78.8/76.4	78.7/73.9	67.7/66.8	79.1/76.8
nsp2	**93.5/91.5**	69.5/65.0	67.6/62.7	67.2/62.4	67.4/62.4	69.5/64.9
nsp3	91.6/**96.5**	**92.0**/96.1	84.6/93.0	86.5/93.5	78.8/88.4	**92.0**/96.1
nsp4	85.9/92.6	**98.5/99.0**	89.2/94.1	91.3/96.1	82.0/91.3	**98.5/99.0**
nsp5	90.2/92.9	98.0/97.6	89.4/93.5	91.8/92.9	81.2/90.7	**98.4/98.2**
nsp6	93.8/**100**	**100/100**	95.8/93.8	93.8/93.8	93.3/93.1	**100/100**
nsp7a	**95.7/96.0**	89.7/**96.0**	85.5/91.9	85.9/93.3	78.4/90.8	89.7/**96.0**
nsp7b	**93.6/95.5**	81.8/79.1	77.0/74.5	78.8/75.5	72.8/71.5	81.8/79.1
nsp8	**94.8/93.3**	89.6/88.9	85.9/88.9	86.7/88.9	78.6/90.5	89.6/88.9
nsp9	90.9/97.7	**96.4/98.9**	90.7/97.3	91.1/97.2	78.6/95.6	**96.4/98.9**
nsp10	87.4/67.0	**98.3**/95.5	89.6/73.4	91.8/79.8	87.2/95.6	**98.3/95.7**
nsp11	**92.4/97.3**	91.6/94.6	90.4/96.4	91.5/96.4	84.1/94.0	92.2/96.0
nsp12	**95.2/94.8**	87.8/91.5	87.8/92.8	88.5/91.5	82.5/90.3	88.0/91.5
GP2a	**94.7/93.4**	87.0/88.3	86.0/86.8	86.3/87.5	84.2/89.7	86.8/87.9
ORF2b	**95.5/93.2**	91.0/87.8	89.6/89.2	89.2/86.5	90.7/89.9	90.5/86.5
GP3	**94.6/91.4**	82.9/79.2	83.5/80.8	83.3/80.8	79.1/80.0	83.0/80.0
GP4	**95.9/96.1**	87.7/87.2	85.8/88.3	87.0/88.3	83.0/86.8	87.7/87.2
GP5	89.3/**89.6**	86.1/84.2	**90.4**/87.6	90.3/88.1	81.3/81.9	86.1/84.2
M	**95.8/97.7**	89.0/92.6	88.4/93.1	87.4/92.0	88.0/92.9	89.1/92.6
N	**96.5/96.8**	90.9/91.1	89.8/89.5	90.9/91.1	85.4/88.8	90.9/91.1
3′UTR	89.4	**92.7**	84.8	85.4	85.3	89.4

Bold face numbers depict the highest percentage identity.

**Table 2:** Information on recombination events of PRRSV isolate, SCN17, detected by RPD4 software.

Recombinant Event	Breakpoints		Major (Similarity)	Minor (Similarity)	*p*-Value of the Detection Methods						
	Beginning	Ending			RDP	GENECONV	BootScan	MaxChi	Chimaera	SiScan	3Seq
1	15	705	NADC30 (94.3%)	RespPRRS MLV (98.4%)	4.370 × 10^−29^	6.623 × 10^−22^	1.109 × 10^−29^	5.911 × 10^−11^	5.319 × 10^−11^	5.136 × 10^−12^	NS
2	4822	6425	NADC30 (94.2%)	RespPRRS MLV (98.7%)	2.367 × 10^−92^	4.075 × 10^−85^	1.104 × 10^−92^	5.846 × 10^−22^	2.225 × 10^−27^	2.942 × 10^−33^	8.000 × 10^−50^
3	7982	10,787	NADC30 (94.2%)	RespPRRS MLV (98.6%)	1.451 × 10^−113^	2.545 × 10^−115^	3.212 × 10^−116^	2.761 × 10^−30^	7.540 × 10^−38^	4.304 × 10^−51^	1.183 × 10^−111^
4	13,604	13,907	NADC30 (94.2%)	JXA1 (96.4%)	7.527 × 10^−23^	3.589 × 10^−12^	2.247 × 10^−23^	2.110 × 10^−08^	1.489 × 10^−09^	2.605 × 10^−06^	NS

NS: Not significant.

## Supplementary Materials

The following are available online at http://www.mdpi.com/1999-4915/10/10/551/s1, Table S1: Information on the 40 reference PRRSVs downloaded from GenBank.
